# Clinical phenotypes and prognosis of cytomegalovirus infection in the pediatric systemic lupus erythematosus: a longitudinal analysis

**DOI:** 10.1186/s12969-023-00807-w

**Published:** 2023-03-16

**Authors:** Tianyu Zhang, Zhongxun Yu, Sihao Gao, Yuelun Zhang, Changyan Wang, Shan Jian, Lin Wang, Lijuan Gou, Ji Li, Mingsheng Ma, Hongmei Song

**Affiliations:** 1 Department of Pediatrics, Peking Union Medical College Hospital, Chinese Academy of Medical Sciences & Peking Union Medical College, No. 1 Shuaifuyuan Wangfujing Dongcheng District, Beijing, 100730 China; 2 Medical Research Center, Peking Union Medical College Hospital, Chinese Academy of Medical Sciences & Peking Union Medical College, Beijing, 100730 China

**Keywords:** Anti-CMV treatment, Clinical phenotypes, Cytomegalovirus infection, Phosphoprotein 65, Systemic lupus erythematosus

## Abstract

**Background:**

Cytomegalovirus (CMV) plays an important role in the pathogenesis of systemic lupus erythematosus (SLE). However, it is not clear whether the anti-CMV treatment has an impact on the prognosis of SLE patients with CMV infection. We aimed to analyze the clinical characteristics and prognosis of CMV infection in pediatric SLE (pSLE) and to evaluate the effect of anti-CMV treatment on pSLE outcome.

**Methods:**

A retrospective study including 146 pSLE from 2012 to 2021 was conducted. CMV-positive and CMV-negative groups were compared by univariate analysis and stepwise logistic multiple regression to analyze the clinical characteristics of CMV infection in pSLE. Generalized estimating equations (GEE) were used to model the longitudinal dynamics of pSLE disease activity with or without CMV infection and anti-CMV treatment.

**Results:**

The CMV infection rate was 74.7% (109/146) in this pSLE cohort. CMV-positive pSLE patients were more likely to present positive anti-dsDNA antibody, hypocomplementemia, high SLEDAI-2K score and musculoskeletal involvement (*P* < 0.05). Survival analysis showed that CMV-positive pSLE patients were more prone to disease flare and poorer outcomes. GEE modeling indicated that CMV phosphoprotein 65 (pp65) titers were positively correlated with SLEDAI-2K, and anti-CMV treatment could better reduce pSLE activity than non-treatment (*P* < 0.05).

**Conclusions:**

CMV infection is highly prevalent among pSLE patients. Positive anti-dsDNA antibody, hypocomplementemia, high SLEDAI-2K score and musculoskeletal involvement were significant clinical clues indicating CMV infections in pSLE. CMV infection is correlated with higher disease activity and poorer outcome. Anti-CMV treatment can reduce disease activity and flares.

**Supplementary Information:**

The online version contains supplementary material available at 10.1186/s12969-023-00807-w.

## Introduction

Systemic lupus erythematosus (SLE) is a chronic autoimmune disease that typically presents with multisystem involvement and positive autoantibodies. Its pathogenesis is complex, and it is generally believed that genetic susceptibility and environmental factors such as viral infection are involved. Although SLE is common in adults, SLE can also affect children, with pediatric SLE (pSLE) accounting for approximately 15-20% of all SLE patients [1]. Incidence rates as high as 0.3-2.5 per 100,000 children per year have been reported, with prevalence rates of 1.89-34.1 per 100,000 children [2].

Cytomegalovirus (CMV) infection is considered to be one of the common complications in SLE patients. Several studies suggest that CMV also plays an important role in the pathogenesis of SLE and may be a predisposing factor for SLE [3, 4]. CMV infection can mimic the onset of SLE and can also lead to severe illness and death [5]. However, it is difficult for clinicians to distinguish whether CMV simulates the onset of SLE or CMV causes the recurrence of SLE and it is not clear whether the anti-CMV treatment has an impact on the prognosis of SLE patients with CMV infection. Our study centered on the clinical features and prognosis of CMV infection in pSLE and shed light on the importance of anti-CMV therapy for rapid remission in SLE.

## Patients and methods

### Study design and patients

This retrospective study was conducted in 146 pSLE patients who were seen and followed up at Peking Union Medical College Hospital (PUMCH) between January 2012 and December 2021. The inclusion criteria were as follows: 1) SLE was diagnosed before the age of 18 and was followed up at PUMCH; 2) patients met the SLE classification criteria of the 1997 American Academy of Rheumatology (ACR) or 2019 European League Against Rheumatism (EULAR)/ACR [6, 7]; 3) Patients completed all three CMV screening tests, including CMV-DNA, CMV- phosphoprotein 65 (pp65), and CMV-IgM. The exclusion criteria were as follows: 1) Evidence of other active infections, such as Epstein-Barr virus, parvovirus B19, bacteria, parasites, chlamydia, mycoplasma, fungi, etc.; 2) Overlap syndrome (overlap between pSLE and other rheumatic diseases); 3) Presence of other diseases such as malignancy and severe malnutrition. We manually set a timeframe of 4 weeks to ensure the consistency of timings between SLE clinical data and CMV infections. Clinical data collected within 4 weeks prior to CMV screening included demographic information, clinical presentation, physical examination, laboratory data, and treatment information. The data of the CMV positive group and the negative group were reviewed and compared. Disease activity was evaluated by the SLE Disease Activity Index-2000 (SLEDAI-2K) [8]. CMV-positive pSLE were divided into treatment and non-treatment groups for comparison and analysis.

### Definition of CMV infection and treatment plan

Many patients were diagnosed and treated in local hospitals, and then transferred to our hospital due to the poor treatment effect or recurrence, so the CMV testing was performed at any time in the SLE process. As long as one or more of the CMV-IgM, CMV-DNA and pp65 antigens in the peripheral blood of pSLE patients are positive, they are considered to be complicated with CMV infection. For pSLE patients who received anti-CMV therapy, the total course of treatment was 3 months, of which the first month received daily intravenous ganciclovir (5 mg/kg, every 12 hours), and the treatment was discontinued after 3 consecutive weeks. Oral ganciclovir (30-50 mg/kg/d) was administered in the first week of the second and third months, respectively.

### Statistical analysis

Data analysis was performed using the Statistical Package for Social Sciences (SPSS) software version 23 and R programming software. Categorical variables were expressed as counts and proportions (%). Continuous variables were expressed as mean ± standard deviation (SD). Univariate analysis using an independent t-test or chi-square test and multivariate analysis using stepwise logistic multiple regression analysis were used to analyze differences between pSLE patients with and without CMV infection and to identify risk factors for pSLE complicated with CMV infection. Associations between CMV infection, anti-CMV therapy, pp65 titers, and disease activity were analyzed using generalized estimating equation (GEE) models. The median difference and odds ratio with their corresponding 95% confidence intervals (CIs) were used to evaluate the effect size between groups. *P* values < 0.05 were interpreted as statistically significant.

## Results

### Demographic and clinical characteristics

As shown in Additional file 1, among the 427 patients with pSLE, 129 were excluded because they did not perform all three CMV tests, and some of them completed one or two tests, of which 83 (64.5%) were CMV positive. Of the remaining children, 20 cases were excluded because of overlap syndrome, 132 cases were excluded because of other active infections, and among them, 116 (87.9%) were CMV positive. Finally, 146 eligible SLE were included, of which 109 (74.7%) were complicated with CMV infection (Additional file 2). Among patients with CMV infection, 54 (49.5%) were positive for CMV IgM antibody, 95 (87.2%) for CMV pp65 antigen, and 11 (10.1%) for CMV-DNA. The average age of pSLE patients was 12.01 ± 2.21 years, and the male to female ratio was 1:3.7. The demographics and clinical presentation of the CMV-positive and negative groups were shown in Table 1. The incidence of fever and musculoskeletal involvement in the positive group was significantly higher than that in the negative group (*P*=0.008). There were no significant differences in age, gender and other clinical manifestations between the two groups. The SLEDAI-2K in the CMV positive group was significantly higher than that in the negative group.

**Table 1:** Demographics and clinical characteristics of pSLE patients (*N =* 146)

**Features**	**CMV positive group** (***N =* 109)**	**CMV negative group** (***N =* 37)**	**OR (95%CI)**	***P* value**
**Demographic Data**				
Age of diagnosis (mean±SD)	11.81±2.07	12.33±1.91	-0.52^a^ (-1.29;0.24)	0.18
Female (%)	87(79.8)	28(75.7)	1.27 (0.53;3.08)	0.595
New-onset (%)	46(42.2)	9(24.3)	2.27 (0.98;5.27)	0.052
**Clinical manifestations**				
Fever (%)	47(43.1)	7(18.9)	3.25 (1.31;8.04)	0.008
Mucocutaneous (%)	71(65.1)	20(54.1)	1.59 (0.75;3.39)	0.229
Musculoskeletal (%)	47(43.1)	7(18.9)	3.25 (1.31;8.04)	0.008
Renal (%)	69(63.3)	21(56.8)	1.31 (0.62;2.81)	0.479
Pleuropulmonary (%)	58(53.2)	16(43.2)	1.49 (0.7;3.16)	0.295
Gastrointestinal (%)	13(11.9)	1(2.7)	4.88 (0.62;38.62)	0.118
Neuropsychiatric (%)	33(30.3)	14(37.8)	0.71 (0.33;1.56)	0.395
Cardiovascular (%)	42(38.5)	8(21.6)	2.27 (0.95;5.44)	0.061
Hematological (%)	87(79.8)	24(64.9)	2.14 (0.94;4.87)	0.066
Endocrine (%)	30(27.5)	11(29.7)	0.9 (0.4;2.04)	0.796

Data are presented as mean ± standard deviation or count (percentage)

*CI* Confidence interval, *CMV* Cytomegalovirus, *OR* Odds ratio, *SLEDAI-2K* Systemic Lupus Erythematosus Disease Activity Index 2000

^a^Median difference

### Laboratory data and treatment

As shown in Table 2, CMV-infected pSLE patients were more prone to hemolytic anemia, elevated liver enzymes, hypoalbuminemia, hypocomplementemia, and multiple autoantibodies including anti-dsDNA antibody, anti-nucleosome antibody, and anti-histone antibody. We observed no significant associations were observed between CMV infection and lymphocyte subsets (Additional file 3).

**Table 2:** Laboratory data of pSLE patients (*N =* 146)

**Clinical features**	**CMV positive group (*N=*109)**	**CMV negative group (*N=*37)**	**OR (95% CI)**	***P* value**
Leukopenia (%)	36 (33)	11 (29.7)	1.17 (0.52;2.62)	0.711
Lymphocytopenia (%)	22 (20.2)	5 (13.5)	1.62 (0.57;4.64)	0.367
Anemia (%)	69 (63.3)	12 (32.4)	3.59 (1.63;7.93)	0.001
Thrombocytopenia (%)	17 (15.6)	6 (16.2)	0.96 (0.35;2.64)	0.929
ALB level<35 g/L(%)	56 (51.4)	9 (24.3)	3.29 (1.42;7.612)	0.004
Elevated ALT (%)	31 (28.4)	2 (5.4)	6.96 (1.58;30.69)	0.004
Elevated AST (%)	33/104 (31.7)	5/36 (13.9)	2.88 (1.03;8.08)	0.038
24h urinary protein ≥ 0.5g (%)	53/96 (55.2)	12/31 (38.7)	1.95 (0.85;4.46)	0.11
Low C3 level (%)	89 (81.7)	12 (32.4)	9.27 (3.99;21.52)	<0.001
Low C4 level (%)	88 (80.7)	11 (29.7)	9.91 (4.23;23.19)	<0.001
Coomb's positivity (%)	55/84 (65.5)	14/32 (43.8)	2.44 (1.06;5.6)	0.033
ANA positivity (%)	107 (98.2)	35 (94.6)	3.06 (0.42;22.52)	0.266
Anti-dsDNA positivity (%)	90 (82.6)	15 (40.5)	6.95 (3.05;15.81)	<0.001
Anti-Sm positivity (%)	34/106 (32.1)	6 (16.2)	2.44 (0.93;6.4)	0.064
Anti-RNP positivity (%)	41/106 (38.7)	10 (27)	1.7 (0.75;3.88)	0.203
Anti-rRNP positivity (%)	37/103 (35.9)	7 (18.9)	2.4 (0.96;6)	0.056
Anti-SSA positivity (%)	46/106 (43.4)	15 (40.5)	1.12 (0.53;2.4)	0.762
Anti-SSB positivity (%)	8/106 (7.5)	4 (10.8)	0.67 (0.19;2.38)	0.508
Anti-Ro52 positivity (%)	15/44 (34.1)	9/23 (39.1)	0.81 (0.28;2.29)	0.683
Anti-histone positivity (%)	29/50 (58)	5/24 (20.8)	5.25 (1.69;16.31)	0.003
Anti-nucleosome positivity (%)	33/50 (66)	6/25 (24)	6.15 (2.07;18.26)	<0.001
Anticardiolipin (%)	18/100 (18)	7/35 (20)	0.88 (0.33;2.32)	0.793
β2GP1(%)	17/103 (16.5)	4/36 (11.1)	1.58 (0.5;5.06)	0.234
Lupus anticoagulant (%)	20/103 (19.4)	12/35 (34.3)	0.46 (0.2;1.08)	0.072

Data are presented as count (percentage)

*ALB* Albumin, *ALT* Alanine aminotransferase, *ANA* Antinuclear antibody, *AST* Aspartate aminotransferase, *β2GP1* Anti-beta2 glycoprotein 1 antibody, *CI* Confidence interval, *CMV* Cytomegalovirus, *OR* Odds ratio

Because immunosuppressive therapy may reduce immune defense in pSLE patients, we collected all patients' medication within 4 weeks before CMV screening and analyzed the effect of medication on CMV infection. 88 patients (60.3%) received prednisone or immunosuppressive therapy, of which hydroxychloroquine was the most commonly used (*N=*56, 38.4%), followed by mycophenolate mofetil (MMF) (*N=* 27, 18.5%) and cyclophosphamide (CTX) (*N=*16, 11%). Only a few patients received cyclosporine (*N=*2), tacrolimus (*N=*2), leflunomide (*N=*5), belimumab (*N=*3), sirolimus (*N=*2) and azathioprine (*N=*1). There was no statistical difference between the CMV-positive group and the CMV-negative group in the dose of prednisone, the percentage of CTX use, or the percentage of glucocorticoid combined with two or more immunosuppressive agents (Additional file 4).

### Multivariate analysis

**Fig. 1:**
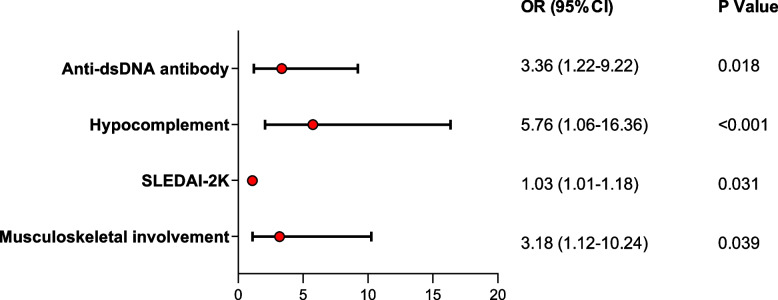
Forest plot of multivariate analysis by stepwise logistic multiple regression. CI, confidence interval; OR, odds ratio; SLEDAI-2K, Systemic Lupus Erythematosus Disease Activity Index 2000

The stepwise logistic multiple regression analysis was then used to further explore the clinical manifestations for CMV infection in pSLE, based on the univariate analysis results. It showed that anti-dsDNA antibody (*P*=0.018, OR 3.36, 95% CI 1.22-9.22), hypocomplementemia (*P*<0.001, OR 5.76, 95% CI 1.06-16.36), SLEDAI-2K score (*P*=0.031, OR 1.03, 95% CI 1.01-1.18), and musculoskeletal involvement (*P*=0.039, OR 3.18, 95% CI 1.12-10.24) were all positively associated with CMV infection in pSLE (Fig. 1).

### Disease activity and outcome

**Fig. 2:**
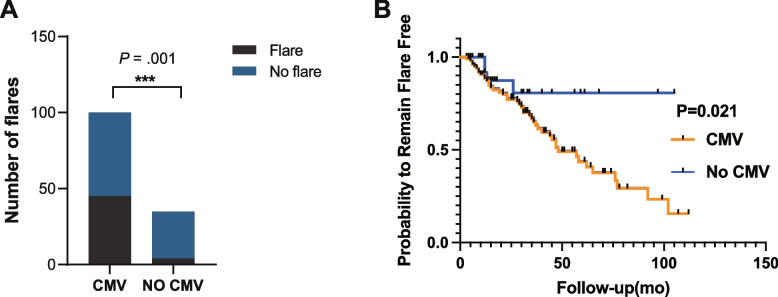
Outcome of the two groups during follow-up. **A** Comparison of flare between the CMV positive group and the negative group. **B** Kaplan-Meier analysis. Kaplan-Meier analysis of overall remission of 101 pSLE with CMV infection and 34 pSLE without CMV infection. CMV, cytomegalovirus; OR, odds ratio

The average SLEDAI-2K was higher in the CMV positive group than that of the CMV negative group [(15.18±7.28) vs (10.22±5.55)] (*P*<0.001). In our longer follow-up study (up to 10 years), 135 patients were followed up consistently. There has been 49 (36.3%) subsequent flare of SLE in the cohort with an average follow-up of 4.32±3.47 years and the CMV-positive group had more cases of subsequent flare (*P*=0.001) (Fig. 2A).

Of the 109 SLE patients with CMV infection, 45 (44.6%) relapsed within a median follow-up period of 47±9 months. Kaplan-Meier analysis showed that SLE patients with CMV infection had a significantly higher risk of progression to flare (*P* = 0.021) (Fig. 2B).

### Prognostic analysis

**Fig. 3:**
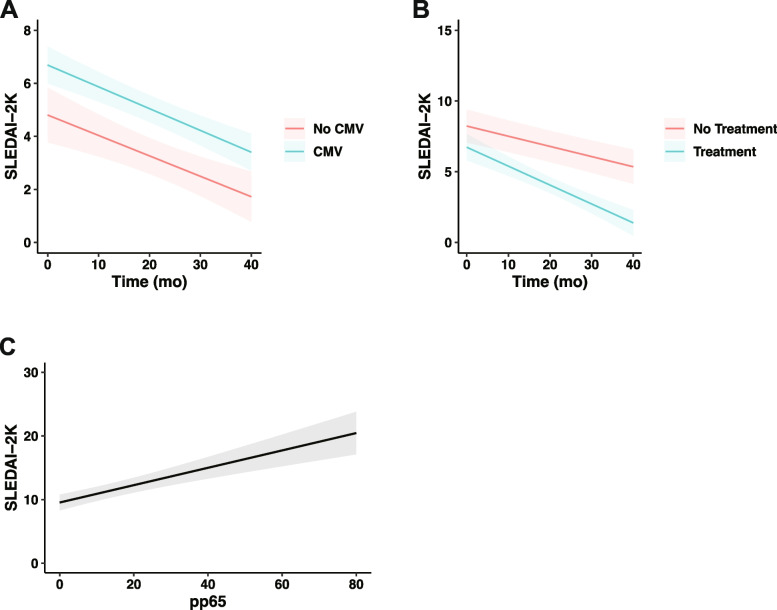
Associations between CMV infection, anti-CMV treatment, pp65 titers and disease activity during follow-up. **A** Association of CMV infection with SLEDAI-2k during follow-up. **B** Association of anti-CMV treatment with SLEDAI-2k during follow-up. **C** Association of pp65 titers with SLEDAI-2K. CMV, cytomegalovirus; SLEDAI-2K, Systemic Lupus Erythematosus Disease Activity Index 2000

Generalized estimating equations were used to evaluate the changes in disease activity over time in the CMV positive and CMV negative groups (Fig. 3A, Table 3), and the results showed that SLEDAI-2K decreased significantly over time in both groups (*P*<0.001). There were significant differences in SLEDAI-2K between the two groups at each follow-up time point (*P*=0.003). The trend of SLEDAI-2K in the two groups over time was similar (*P*=0.817).

**Table 3:** Generalized estimating equation models predicting SLEDAI-2K with CMV infection, anti-CMV treatment and pp65 titers during follow-up

**Outcome: SLEDAI-2K**	**Beta coefficient (95%CI)**	***P* value**
**Model 1: CMV vs. No CMV over time**		
Time	-0.08(-0.11;-0.04)	<0.001
No CMV	1(ref)	ref
CMV	1.88(0.64;3.13)	0.003
No CMV*Time	1(ref)	ref
CMV*Time	-0.01(-0.05;0.04)	0.817
**Model 2: Anti-CMV treatment vs. No treatment over time**		
Time	-0.07(-0.09;-0.05)	<0.001
No Treatment	1(ref)	ref
Treatment	-1.50(-2.97;-0.02)	0.046
No Treatment*Time	1(ref)	ref
Treatment*Time	-0.06(-0.10;-0.02)	0.001
**Model 3: pp65**		
pp65	0.14(0.09;0.18)	<0.001

Beta coefficient for ‘time’ represents the change of SLEDAI-2K over the follow-up period

*CI* Confidence interval, *SLEDAI-2K* Systemic Lupus Erythematosus Disease Activity Index 2000

* indicates the interaction between two variables

The infection group was further divided into the treatment group and the non-treatment group. The generalized estimating equation was used to evaluate the changes in disease activity in the two groups over time (Fig. 3B, Table 3). The results showed that SLEDAI-2K decreased significantly in both groups over time (*P*<0.001). There were significant differences in SLEDAI-2K between the two groups at each follow-up time point (*P*=0.046). There was a significant difference in the trend of SLEDAI-2K between the two groups over time (*P*=0.001).

Generalized estimating equations were used to evaluate the relationship between pp65 titer and SLEDAI, and the results showed a positive correlation (*P* <0.001) (Fig. 3C, Table 3).

## Discussion

CMV is a beta-herpesvirus that humans usually acquire in childhood and is often self-limited after infection. CMV infection has a diffuse presentation that may resemble an episode of disease that complicates the clinical presentation of SLE. Our study analyzed the clinical characteristics of CMV infection in pSLE and demonstrated that anti-CMV treatment may induce faster disease remission in pSLE population.

We proposed the CMV infection rate is high among patients with pSLE and monitoring pp65 in pSLE patients with CMV infection is important. CMV seroprevalence in adults is approximately 45% to 100%. In a study in which CMV infection was diagnosed by virus isolation, the prevalence of CMV infection at the time of diagnosis of pSLE was 1.04% [9]. In our study, the prevalence of CMV infection in pSLE was 74.7% (109/146). Differences in CMV positivity rates between studies may be due to different definitions of CMV infection. Previous studies [10–16] demonstrated that CMV antigenemia was a sensitive, specific and rapid indicator for the early diagnosis of CMV infection. The prevalence of CMV antigenemia in patients with SLE (58.6%) was much higher than in patients with non-SLE autoimmune diseases, especially in patients with pSLE [17]. In our study, CMV infection was primarily diagnosed by CMV antigenemia, and pSLE disease activity was positively correlated with pp65 titers. These conclusions support the theory that CMV pp65 may play a significant role in triggering SLE [18] and CMV pp65 positive appear in the early stage of viral infection activation, which is helpful for the early diagnosis of CMV infection, so suggesting the importance of screening and monitoring pp65 in pSLE patients to facilitate early diagnosis and treatment of CMV infection.

Our study provided some clinical characteristics of pSLE with CMV infection. Previous studies [19] have shown that hematological abnormalities are the most common manifestations of rheumatic diseases complicated by CMV infection, followed by hepatitis, pneumonia, and gastrointestinal damage. Although some results of the univariate analysis were similar to the previous results, stepwise logistic multiple regression analysis showed that CMV infection in pSLE was more likely to be associated with positive anti-dsDNA antibody, hypocomplementemia, high SLEDAI-2K score and musculoskeletal involvement. Previous studies [20, 21] and our study showed that CMV infection may play a role in the initiation or amplification of inflammatory responses in arthritis, though findings offer little insight into potential mechanisms. Higher rates of immunosuppressive therapy, pulse steroid therapy, and CTX use had been reported in CMV-infected SLE patients compared with uninfected SLE patients [9, 12, 22]. However, our study and another study showed no significant differences in steroid dose, CTX, and use of immunosuppressive therapy between CMV-positive and negative groups [23]. Therefore, the risk factors of CMV infection need to be further studied. In conclusion, positive anti-dsDNA antibody, hypocomplementemia, high SLEDAI-2K score and musculoskeletal involvement may be important clues to CMV infection in pSLE patients.

The association between CMV infection and disease activity of SLE is still controversial, and data on the impact of CMV infection on pSLE prognosis are limited. In adult studies, Wu CS et al. [24] reported that SLE patients with CMV infection were associated with lower disease activity while another study showed the severity of clinical features and SLEDAI scores were considerably higher in SLE patients with CMV infection than in SLE patients without CMV infection [25]. In pediatric study, Lee PP et al. [26] reported that repeated CMV infection was associated with poor SLE outcomes. Our study showed that in pSLE patients, the SLEDAI-2K score and recurrence rate was higher in the CMV-positive group than in the negative group. In addition, the proportion of hypocomplementemia and anti-dsDNA antibody positivity in the CMV-positive group was significantly higher than that in the CMV-negative group, which may explain why SLE patients in the CMV-positive group had higher SLEDAI-2K scores. Previous animal studies had shown that CMV pp65 induced cross-reactivity to dsDNA and lead to renal histological damage [27, 28]. In our study, however, the incidence of lupus nephritis was not significantly increased in the CMV-positive group (63.3% vs 56.8%).

Our study also suggested that anti-CMV therapy is important in pSLE patients. In this retrospective study spanning 10 years, CMV positive patients did not remain positive over the course of the study. Not all pSLE with CMV infection received anti-CMV therapy, most patients turned negative after antiviral treatment, while a small number remained positive or turned negative without treatment. We found that, although the non-treatment group had slightly higher SLEDAI-2K scores than the treatment group, the trend of decreasing SLEDAI-2K in the treatment group was more pronounced over time. This showed that the combination of anti-CMV therapy on the basis of standard SLE treatment could accelerate the clinical remission of pSLE complicated by CMV infection.

Our study has some limitations. First, this is a single-center retrospective study, the CMV testing was performed at any time in the SLE process, so it is not clear the temporal relationship between pSLE and CMV infection, and despite reviewing all cases diagnosed with SLE, there may still be bias. In addition, the sample size of pSLE without CMV infection is relatively small, and the conclusions drawn from the comparative analysis with the cohort of pSLE infected with CMV still need to be applied with caution. Therefore, prospective studies with larger sample sizes are still needed to further validate these findings in the future.

## Conclusions

CMV infection rate was high in pSLE, and pSLE patients with CMV infection had higher disease activity and were more likely to have anti-dsDNA antibody, hypocomplementemia, and musculoskeletal involvement. The prognosis of the CMV-positive patients was relatively poor, especially in those who did not receive anti-CMV treatment. Therefore, early identification and treatment of CMV infection are very important to improve patient outcomes. In patients with CMV-positive pSLE, CMV-pp65 titers should be monitored. Prevention and monitoring of CMV infection is an important part of the management of pSLE patients.

## Supplementary Information

**Additional file 1. **Flow diagram for the study.

**Additional file 2. **CMV-related test results in CMV-positive pSLE.

**Additional file 3. **Distribution of lymphocyte subsets in SLE patients with and without infection.

**Additional file 4. **Treatment for SLE within 4 weeks prior to CMV screening tests.

## Data Availability

Data are available on reasonable request. The datasets generated during and/or analysed during the current study are available from the corresponding author on reasonable request.

## Abbreviations

ALB: Albumin
ACR: American Academy of Rheumatology
ALT: Alanine aminotransferase
ANA: Antinuclear antibody
AST: Aspartate aminotransferase
CMV: Cytomegalovirus
CIs: Confidence intervals
CTX: Cyclophosphamide
EULAR: European League Against Rheumatism
GEE: Generalized estimating equations
MMF: Mycophenolate mofetil
OR: Odds ratio
Pp65: Phosphoprotein 65
PUMCH: Peking Union Medical College Hospital
SD: Standard deviation
SLE: Systemic Lupus Erythematosus
SLEDAI-2K: SLE Disease Activity Index-2000
SPSS: Statistical Package for Social Sciences
β2GP1: Anti-beta2 glycoprotein 1 antibody
