# Supplementary material for: Clinical phenotypes and prognosis of cytomegalovirus infection in the pediatric systemic lupus erythematosus: a longitudinal analysis
Source: Pediatr Rheumatol Online J. 2023 Mar 16;21:25. doi: 10.1186/s12969-023-00807-w (PMC10022138; doi:10.1186/s12969-023-00807-w)
Supplement: Supplementary file 1 — Additional file 1. Flow diagram for the study. [file 12969_2023_807_MOESM1_ESM.docx]

**Additional file 1** | Flow diagram for the study

CMV: cytomegalovirus.
