# Supplementary figures and images for: Clinical phenotypes and prognosis of cytomegalovirus infection in the pediatric systemic lupus erythematosus: a longitudinal analysis
Source: Pediatr Rheumatol Online J. 2023 Mar 16;21:25. doi: 10.1186/s12969-023-00807-w (PMC10022138; doi:10.1186/s12969-023-00807-w)

**Additional file 2 |** CMV-related test results in CMV-positive pSLE.


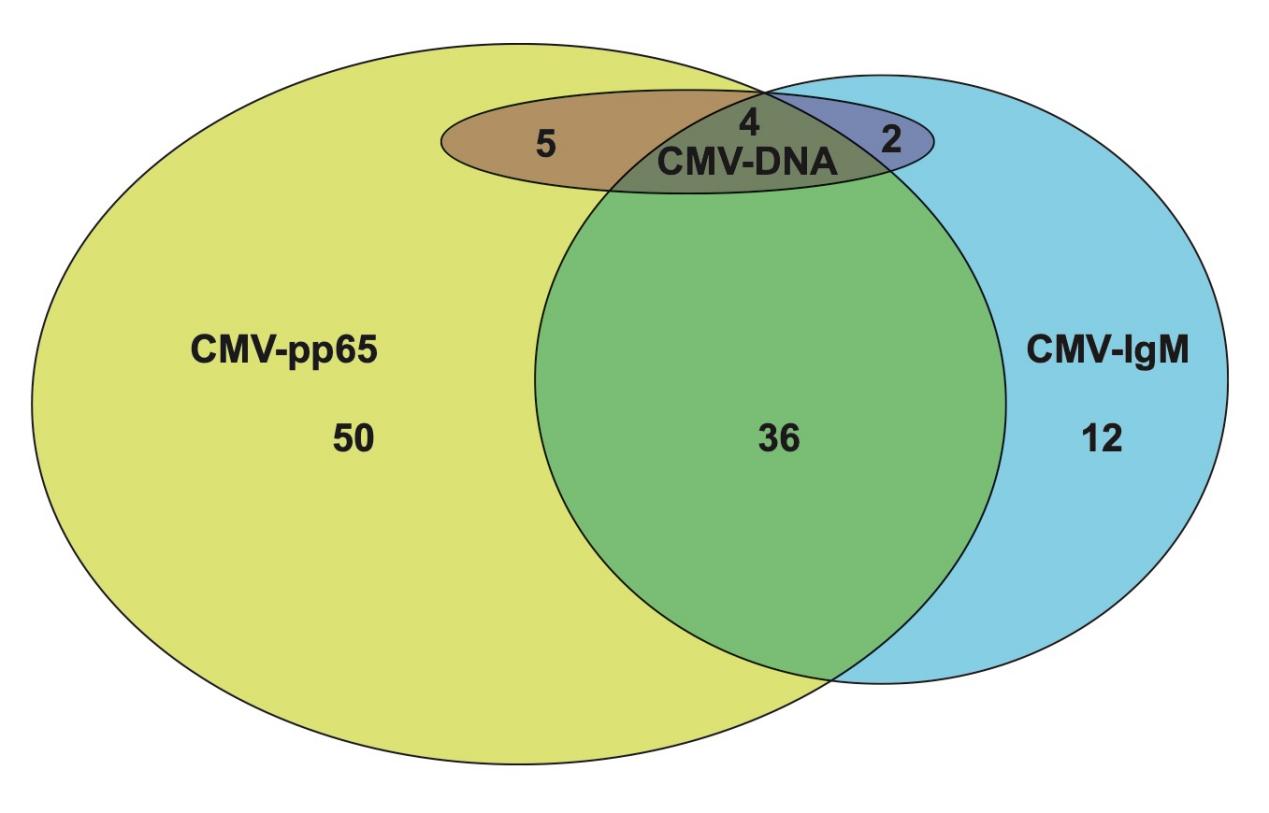


CMV: cytomegalovirus.

Supplement: Supplementary file 2 — Additional file 2. CMV-related test results in CMV-positive pSLE. [file 12969_2023_807_MOESM2_ESM.docx]
