# Supplementary material for: Clinical phenotypes and prognosis of cytomegalovirus infection in the pediatric systemic lupus erythematosus: a longitudinal analysis
Source: Pediatr Rheumatol Online J. 2023 Mar 16;21:25. doi: 10.1186/s12969-023-00807-w (PMC10022138; doi:10.1186/s12969-023-00807-w)
Supplement: Supplementary file 3 — Additional file 3. Distribution of lymphocyte subsets in SLE patients with and without infection. [file 12969_2023_807_MOESM3_ESM.docx]

**Additional file 3 |** Distribution of lymphocyte subsets in SLE patients with and without infection.

| **Features** | **CMV positive group (N=109)** | **CMV negative group (N=37)** | ***P* value** |
| --- | --- | --- | --- |
| Lymphocyte (/uL) | 1618.1±1140.59 | 1891.14±2147.06 | 0.737 |
| CD3+ (%) | 74.93±9.54 | 76.34±6.31 | 0.778 |
| CD4+T (%) | 35.54±9.07 | 36.67±7.51 | 0.258 |
| CD8+T (%) | 48.43±91.17 | 33.95±7.16 | 0.682 |
| CD19+ (%) | 18.92±10.47 | 18.47±7.1 | 0.315 |
| CD16+CD56+ (%) | 5.46±5.34 | 3.49±1.89 | 0.139 |
| CD3+ (/μL) | 1078.78±630.10 | 1403.43±1502.7 | 0.787 |
| CD4+T (/μL) | 509.18±334.16 | 719.86±895.57 | 0.939 |
| CD8+T (/μL) | 491.38±295.39 | 559.43±454.84 | 0.666 |
| CD19+ (/μL) | 320.35±573.08 | 400±584.12 | 0.879 |
| CD16+CD56+ (/μL) | 65.46±61.13 | 60.43±50.14 | 0.067 |
| CD4/CD8 | 1.1±0.45 | 1.15±0.45 | 0.233 |

CTX: Cyclophosphamide
