# Supplementary material for: Clinical phenotypes and prognosis of cytomegalovirus infection in the pediatric systemic lupus erythematosus: a longitudinal analysis
Source: Pediatr Rheumatol Online J. 2023 Mar 16;21:25. doi: 10.1186/s12969-023-00807-w (PMC10022138; doi:10.1186/s12969-023-00807-w)
Supplement: Supplementary file 4 — Additional file 4. Treatment for SLE within 4 weeks prior to CMV screening tests. [file 12969_2023_807_MOESM4_ESM.docx]

**Additional file 4** | Treatment for SLE within 4 weeks prior to CMV screening tests.

| **Features** | **CMV positive group (N=109)** | **CMV negative group (N=37)** | ***P* value** |
| --- | --- | --- | --- |
| Prednisone | 55 (50.5) | 27 (73) | 0.423 |
| Average dose of prednisone |  |  | 0.652 |
| 0＜ and ≤0.5mg/kg | 15 (13.8) | 10 (27) |  |
| 0.5＜ and ≤1mg/kg | 13 (11.9) | 6 (16.2) |  |
| 1＜ and ≤2mg/kg | 27 (24.8) | 11 (29.7) |  |
| Pulse glucocorticoid | 17 (15.7) | 6 (16.2) | 0.946 |
| CTX | 12 (11) | 5 (13.5) | 0.767 |
| Glucocorticoid combined with 2 or more Immunosuppressive drugs | 24 (22.2) | 11 (29.7) | 0.357 |

CMV: cytomegalovirus.
